# Space electric field concentrated effect for Zr:SiO_2_ RRAM devices using porous SiO_2_ buffer layer

**DOI:** 10.1186/1556-276X-8-523

**Published:** 2013-12-11

**Authors:** Kuan-Chang Chang, Jen-wei Huang, Ting-Chang Chang, Tsung-Ming Tsai, Kai-Huang Chen, Tai-Fa Young, Jung-Hui Chen, Rui Zhang, Jen-Chung Lou, Syuan-Yong Huang, Yin-Chih Pan, Hui-Chun Huang, Yong-En Syu, Der-Shin Gan, Ding-Hua Bao, Simon M Sze

**Affiliations:** 1 Department of Materials and Optoelectronic Science, National Sun Yat-Sen University, Kaohsiung, Taiwan; 2 Department of Physics, R.O.C. Military Academy, Kaohsiung, Taiwan; 3 Department of Physics, National Sun Yat-Sen University, Kaohsiung 804, Taiwan; 4 Advanced Optoelectronics Technology Center, National Cheng Kung University, Tainan, Taiwan; 5 Department of Electronics Engineering and Computer Science, Tung Fang Design Institute, Kaohsiung, Taiwan; 6 Department of Mechanical and Electro-Mechanical Engineering, National Sun Yat-sen University, Kaohsiung, Taiwan; 7 Department of Chemistry, National Kaohsiung Normal University, Kaohsiung, Taiwan; 8 School of Software and Microelectronics, Peking University, Beijing, People’s Republic of China; 9 State Key Laboratory of Optoelectronic Materials and Technologies, School of Physics and Engineering, Sun Yat-Sen University, 510275, Guangzhou, China; 10 Department of Electronics Engineering and Institute of Electronics, National Chiao Tung University, Hsinchu, Taiwan

**Keywords:** RRAM, Porous SiO_2_, Space charge limited current, Zr

## Abstract

To improve the operation current lowing of the Zr:SiO_2_ RRAM devices, a space electric field concentrated effect established by the porous SiO_2_ buffer layer was investigated and found in this study. The resistive switching properties of the low-resistance state (LRS) and high-resistance state (HRS) in resistive random access memory (RRAM) devices for the single-layer Zr:SiO_2_ and bilayer Zr:SiO_2_/porous SiO_2_ thin films were analyzed and discussed. In addition, the original space charge limited current (SCLC) conduction mechanism in LRS and HRS of the RRAM devices using bilayer Zr:SiO_2_/porous SiO_2_ thin films was found. Finally, a space electric field concentrated effect in the bilayer Zr:SiO_2_/porous SiO_2_ RRAM devices was also explained and verified by the COMSOL Multiphysics simulation model.

## Background

Recently, various non-volatile random access memory (NvRAM) such as magnetic random access memory (MRAM), ferroelectric random access memory (FeRAM), phrase change memory (PCM), and resistive random access memory (RRAM) were widely investigated and discussed for applications in portable electronic products which consisted of low power consumption IC [1], non-volatile memory [2-6], and TFT LCD display [7-10]. To overcome the technical and physical limitation issues of conventional charge storage-based memories [11-18], the resistive random access memory (RRAM) device which consisted of the oxide-based layer sandwiched by two electrodes was a great potential candidate for the next-generation non-volatile memory because of its superior properties such as low cost, simple structure, fast operation speed, low operation power, and non-destructive readout properties [19-42].

In our previous report, the resistive switching stability and reliability of RRAM device can be improved using a high/low permittivity bilayer structure [43]. Because the permittivity of porous SiO_2_ film is lower than that of SiO_2_ film, the zirconium metal doped into SiO_2_ (Zr:SiO_2_) thin film fabricated by co-sputtering technology and the porous SiO_2_ buffer layer prepared by inductively coupled plasma (ICP) treatment were executed to form Zr:SiO_2_/porous SiO_2_ RRAM devices in this study. In addition, the resistive switching behaviors of the Zr:SiO_2_ RRAM devices using the bilayer structure were improved and investigated by a space electric field concentrated effect.

## Methods

To generate a space electric field concentrated effect in RRAM devices, the porous SiO_2_ buffer layer in the bilayer Zr:SiO_2_/porous SiO_2_ structure was proposed. The patterned TiN/Ti/SiO_2_/Si substrate was obtained by standard deposition and etching process; after which, 1 μm × 1 μm via holes were formed. After that, the C:SiO_2_ film was prepared by co-depositing with the pure SiO_2_ and carbon targets, and the porous SiO_2_ thin film (about 6 nm) was formed by ICP O_2_ plasma technology. Then, the Zr:SiO_2_ thin film (about 20 nm) was deposited on the porous SiO_2_ thin film by co-sputtering with the pure SiO_2_ and zirconium targets. The sputtering power was fixed with rf power 200 W and direct current (DC) power 10 W for silicon dioxide and zirconium targets, respectively. A Pt electrode of 200-nm thickness was deposited on all samples by DC magnetron sputtering. Finally, all electrical devices were fabricated through lithography and lift-off techniques. Besides, the Fourier transform infrared spectroscopy (FTIR) was used to analyze the chemical composition and bonding of the Zr:SiO_2_ thin films, and the entire electrical measurements of devices with the Pt electrode were performed using Agilent B1500 semiconductor parameter analyzer (Santa Clara, CA, USA).

## Results and discussion

**Figure 1:**
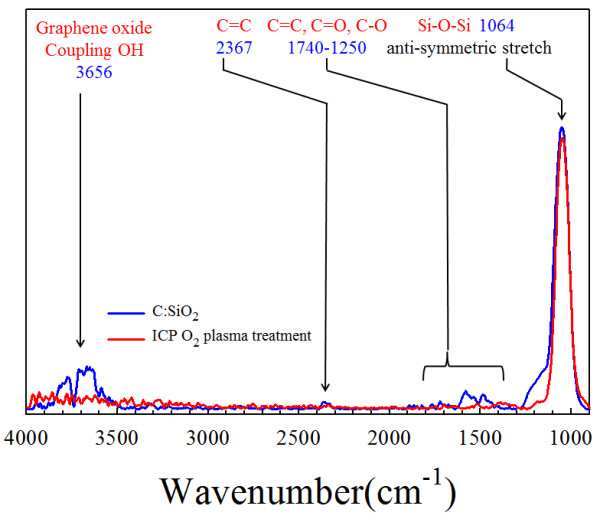
**Comparison of FTIR spectra of the C:SiO**_**2 **_**thin film before and after oxygen plasma treatment.**

To verify the porous SiO_2_ layer generated and formed, the FTIR spectra of the non-treated and treated C:SiO_2_ thin film prepared by the oxygen plasma treatment was compared and showed in Figure 1. It was clearly observed that the absorption of anti-symmetric stretch mode of Si-O-Si bonding was at 1,064 cm^-1^ in the non-treated and treated C:SiO_2_ thin film by oxygen plasma treatment. In addition, the C = C bonding at 2,367 cm^-1^, C:SiO_2_ coupling OH bonding at 3,656 cm^-1^, C-O bonding, and C-C bonding from 1,250 to 1,740 cm^-1^ were found. This result implicated that the porous SiO_2_ thin film was formed by the chemical reaction between carbon and oxygen plasma treatment.

**Figure 2:**
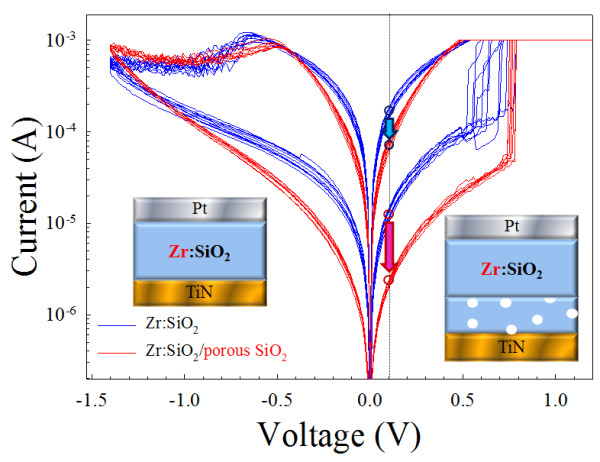
**Current–voltage curves and the resistive switching characteristics of Zr:SiO**_**2 **_**and bilayer Zr:SiO**_**2**_**/porous SiO**_**2 **_**RRAM devices.** The schematic configuration of the Zr:SiO_2_ RRAM and bilayer Zr:SiO_2_/porous SiO_2_ RRAM in the inset of the figure.

The forming process for the compliance current of 1 μA was required to activate all of the single-layer Zr:SiO_2_ and bilayer Zr:SiO_2_/porous SiO_2_ thin film RRAM devices. For Zr:SiO_2_ RRAM devices, the sweeping voltage was applied on TiN electrode with the grounded Pt electrode. Figure 2 shows the resistive switching characteristics of the single-layer Zr:SiO_2_ and the bilayer Zr:SiO_2_/porous SiO_2_ RRAM devices, respectively. The single-layer Zr:SiO_2_ and the bilayer Zr:SiO_2_/porous SiO_2_ RRAM device structure were also shown in the inset of Figure 2. At the reading voltage of 0.1 V, the operation current of the LRS and HRS in Zr:SiO_2_ RRAM devices using the porous SiO_2_ buffer layer was smaller than that of others. A space electric field concentrated effect was testified to cause the operation current lowing of the RRAM devices using the porous SiO_2_ buffer layer.

**Figure 3:**
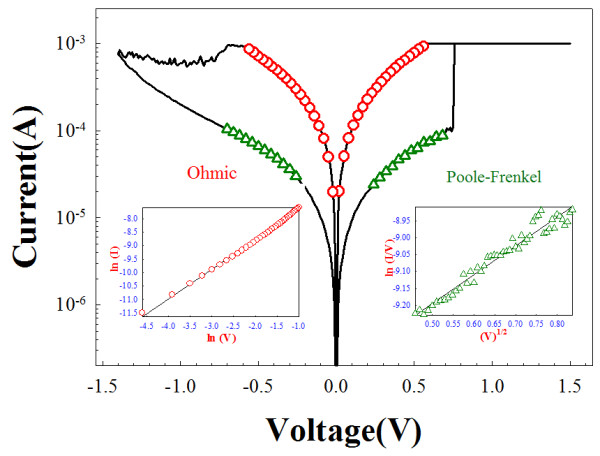
**Carrier transport analyzed for LRS and HRS of the Zr:SiO2 RRAM by the curve fitting.** The carrier transport analyzed in conduction mechanism for LRS and HRS of the single-layer Zr:SiO_2_ RRAM devices by the curve fitting.

**Figure 4:**
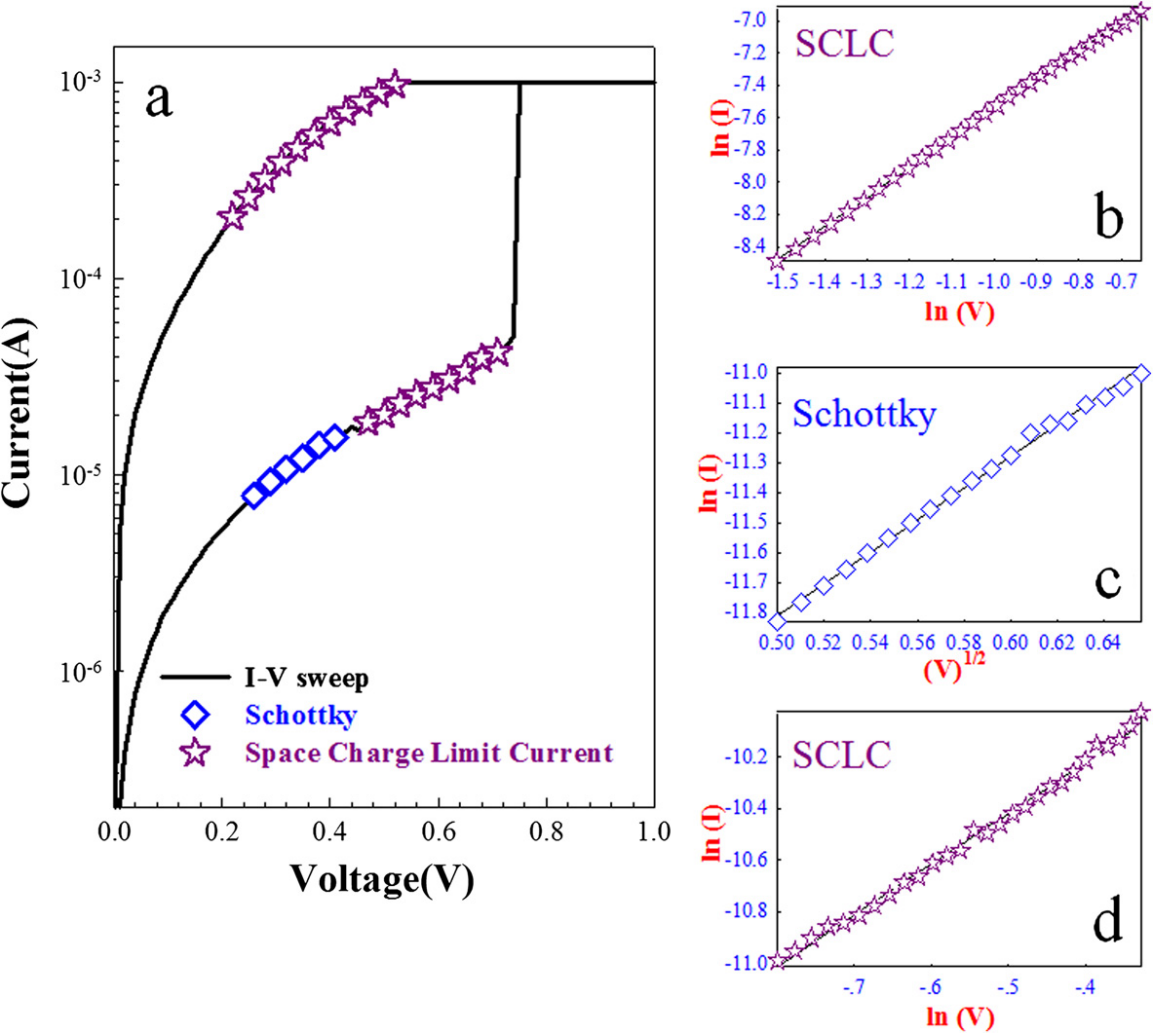
**Carrier transport and *I*-*V *plots. (a)** The carrier transport analyzed in conduction mechanism for LRS and HRS of the single bilayer Zr:SiO_2_/porous SiO_2_ RRAM devices by the curve fitting. **(b)** In (*I*-*V*), **(c)** In (*I*-*V*^1/2^), and **(d)** In (*I*-*V*) plots.

In order to further discuss the resistive switching mechanism in single-layer Zr:SiO_2_ and bilayer Zr:SiO_2_/porous SiO_2_ RRAM devices, the conduction mechanism of current–voltage (*I*-*V*) curves in LRS and HRS were analyzed to discuss the carrier transport in the switching layer in Figures 3 and 4. The carrier transport of the LRS in Zr:SiO_2_ RRAM devices dominated by ohmic conduction mechanism is shown in the left inset of Figure 3. The result revealed that the conductive filament formed by the defect is induced by the zirconium atoms as the current flows through the Zr:SiO_2_ film. As shown in the right inset of Figure 3, the carrier transport in HRS of Zr:SiO_2_ RRAM was dominated by Pool-Frenkel emission, which resulted from the thermal emission of trapped electrons in the Zr:SiO_2_ film. However, for the bilayer Zr:SiO_2_/porous SiO_2_ structure, the current mechanism of the LRS in Zr:SiO_2_ RRAM devices was dominated by the space charge limited current (SCLC) conduction (Figure 4b). Additionally, the current conduction mechanism of the HRS in Zr:SiO_2_/porous SiO_2_ RRAM devices was transferred from Schottky emission to SCLC conduction in Figure 4c,d. These results indicated that the filament is connected to the pore of porous SiO_2_ film after the forming process and the SCLC conduction mechanism is caused by an electric field concentrated effect.

**Figure 5:**
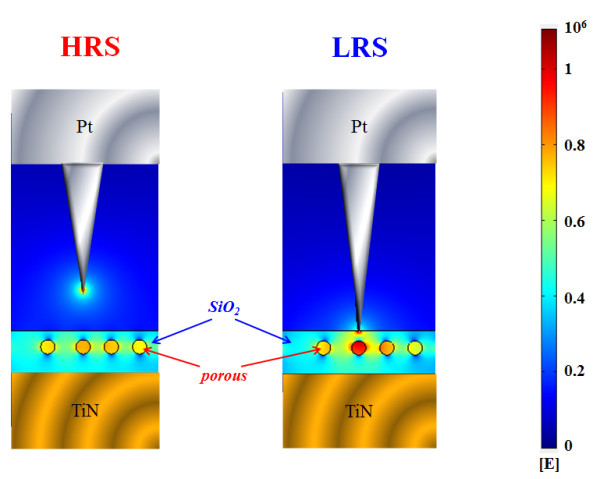
**Electric field simulation in LRS and HRS for Pt/Zr:SiO**_**2**_**/porous SiO**_**2**_**/TiN RRAM devices.**

To clarify and discuss the SCLC conduction mechanism in bilayer Zr:SiO_2_/porous SiO_2_ RRAM devices, the COMSOL Multiphysics simulation model was employed to analyze the distribution of electric field concentrated effect. Figure 5 shows the distribution of the electric field in the bilayer Zr:SiO_2_/porous SiO_2_ RRAM devices for LRS and HRS. A high density of electric field exists in and around the area of the pore in porous SiO_2_ film, which confirms the electric field concentrating capability of nanopores. Thus, during the set process, the metal conduction filament has an inclination to form towards the direction of the pore, and the conduction of the electron was dominated by the SCLC conduction in the porous SiO_2_ film.

## Conclusion

In conclusion, a space electric field concentrated effect was demonstrated to cause the operation current lowing for the Zr:SiO_2_ RRAM devices. In addition, the single-layer Zr:SiO_2_ and bilayer Zr:SiO_2_/porous SiO_2_ were prepared to investigate the resistive switching characteristics of RRAM devices. Compared with the conduction mechanism of the bilayer Zr:SiO_2_/porous SiO_2_ RRAM with single-layer Zr:SiO_2_ RRAM, the conduction mechanism of the LRS was transferred from ohmic to SCLC conduction mechanism. Besides, the conduction mechanism of the HRS was transferred from Pool-Frenkel emission to Schottky emission at low field and dominated by SCLC at high field. Through a space electric field concentrated effect, the SCLC conduction of the Zr:SiO_2_ RRAM devices using the porous SiO_2_ buffer layer was explained and discussed by the COMSOL Multiphysics simulation model.

## Competing interests

The authors declare that they have no competing interests.

## Authors’ contributions

K-CC designed and set up the experimental procedure. J-WH and T-CC planned the experiments and agreed with the paper's publication. T-MT, K-HC, T-FY, J-HC, D-SG, and J-CL revised the manuscript critically and made some changes. RZ fabricated the devices with the assistance of S-YH. Y-CP conducted the electrical measurement of the devices. H-CH and Y-ES performed the FTIR spectra measurement. SMS and DHB assisted in the data analysis. All authors read and approved the final manuscript.
